# A Genome-Wide Association Study Identified Novel Genetic Susceptibility Loci for Oral Cancer in Taiwan

**DOI:** 10.3390/ijms24032789

**Published:** 2023-02-01

**Authors:** Da-Tian Bau, Ting-Yuan Liu, Chia-Wen Tsai, Wen-Shin Chang, Jian Gu, Jai-Sing Yang, Liang-Chun Shih, Fuu-Jen Tsai

**Affiliations:** 1 Graduate Institute of Biomedical Sciences, China Medical University, Taichung 404333, Taiwan; 2 Terry Fox Cancer Research Laboratory, Department of Medical Research, China Medical University Hospital, Taichung 404327, Taiwan; 3 Department of Bioinformatics and Medical Engineering, Asia University, Taichung 41354, Taiwan; 4 Department of Medical Research, China Medical University Hospital, China Medical University, Taichung 404327, Taiwan; 5 Department of Epidemiology, The University of Texas MD Anderson Cancer Center, Houston, TX 77030, USA; 6 Human Genetics Center, Department of Medical Research, China Medical University Hospital, Taichung 404327, Taiwan; 7 Department of Medical Genetics, China Medical University Hospital, Taichung 404327, Taiwan

**Keywords:** oral cancer, genome-wide association study, SNP, HLA, TERT, TPRS1, TMED3

## Abstract

Taiwan has the highest incidence rate of oral cancer in the world. Although oral cancer is mostly an environmentally induced cancer, genetic factors also play an important role in its etiology. Genome-wide association studies (GWAS) have identified nine susceptibility regions for oral cancers in populations of European descent. In this study, we performed the first GWAS of oral cancer in Taiwan with 1529 cases and 44,572 controls. We confirmed two previously reported loci on the 6p21.33 (HLA-B) and 6p21.32 (HLA-DQ gene cluster) loci, highlighting the importance of the human leukocyte antigen and, hence, the immunologic mechanisms in oral carcinogenesis. The TERT-CLMPT1L locus on 5p15.33, the 4q23 ADH1B locus, and the LAMC3 locus on 9q34.12 were also consistent in the Taiwanese. We found two new independent loci on 6p21.32, rs401775 in SKIV2L gene and rs9267798 in TNXB gene. We also found two suggestive novel Taiwanese-specific loci near the TPRS1 gene on 8q23.3 and in the TMED3 gene on 15q25.1. This study identified both common and unique oral cancer susceptibility loci in the Taiwanese as compared to populations of European descent and shed significant light on the etiology of oral cancer in Taiwan.

## 1. Introduction

Oral cancer is the eighth most common cancer in men worldwide with an estimated 264,211 new cases in men in 2020 [1]. The age-standardized incidence rate was 2.6-fold higher in men (6.0 per 100,000) than in women (2.3 per 100,000) worldwide, although the rate varies widely across regions, with a 20-fold difference in men and 10-fold difference in women [1]. Taiwan has the highest incidence rate of oral cancer in men (27.01 per 100,000) in the world and the male predominance is even more pronounced with nearly 90% of oral cancers occurring in men, accounting for the third highest incidence rate and fourth highest cause of cancer deaths among all cancers among Taiwanese men [2]. The high incidence rate and striking male predominance of oral cancer in Taiwan are attributed to the high prevalence of major risk factors, including smoking, alcohol drinking, and particularly, betel quid chewing [2,3,4,5]. Betel quid chewing confers an approximately 8-fold increased risk of oral cancer, much higher that the risks conferred by smoking (3.6-fold) and drinking (2.2-fold) [6]. More significantly, there is a strong synergistic effect of smoking, drinking, and chewing in promoting oral carcinogenesis, resulting in an over 40-fold increased oral cancer risk for the smoking–drinking–chewing persons [6,7,8].

Although oral cancer is mostly an environmentally induced cancer, genetic factors and gene–environment interactions also play an important role in its etiology. Epidemiological studies have observed an elevated risk of oral cancer in individuals with a family history of head and neck cancer [9,10,11]. Numerous candidate gene studies have been performed to assess the associations of selected single nucleotide polymorphisms (SNPs) in genes involved in essential biological pathways such as carcinogen metabolism, DNA repair, cell-cycle control, and inflammatory response with the risks of head and neck cancer overall and/or oral cancer specifically [12,13,14,15,16,17,18,19,20,21,22]. The only consistent associations from numerous candidate gene studies are those related to alcohol dehydrogenase (ADH) genes: SNPs in several ADH genes were associated with the risks of upper aerodigestive cancers including oral cancer and the effects became more apparent with increasing alcohol consumption, indicating a gene–environment interaction [20,23]. The genome-wide association study (GWAS) has revolutionized genetic association research and identified hundreds of thousands of novel susceptibility loci for hundreds of genetic traits, including approximately 800 susceptibility loci for over 20 different cancers [24,25,26]. There were only three published GWAS of head and neck cancer or upper aerodigestive cancer that included the subtype of oral cancer, all in European populations [23,27,28]. These GWAS identified at least nine genetic susceptibility regions for oral cancers, including 2p23.3 (GPN1), 4q21 (HEL308 and FAM175A), 4q23 (ADH1B, ADH1C, ADH7), 5p15.33 (CLPTM1L), 6p21 (HLA), 6p22.1 (ZNRD1-AS1), 9p21.3 (CDKN2A–CDKN2B), 9q34.12 (LAMC3), and 12q24 (ALDH2). These loci only explain a small portion of the genetic heritability of oral cancer. More susceptibility loci remain to be discovered. Furthermore, these loci were found in European descendants, who have much a lower incidence rate than Taiwan and were not exposed to betel quid. No GWAS of oral cancer has been conducted in Taiwan. Given the common (smoking and alcohol drinking) and distinct environmental exposures (betel quid chewing) in Taiwan, and the diversity of genetic structure across different ethnicities, we hypothesize that there are common and unique genetic susceptibility loci of oral cancer in the Taiwanese as compared with the European population. We therefore performed the first GWAS of oral cancer in Taiwan.

## 2. Results

### 2.1. Demographics of Study Population

After strict quality control procedures, data from 1529 cases and 44,572 controls were included in the final analysis. Table 1 shows the selected characteristics of the cases and controls. The mean ages (standard deviation) of the cases and controls were 55 (11.5) and 54 (14.5) years, respectively. About 86.9% (N = 1328) of the patients and 77.7% (N = 34,616) of the controls were men.

### 2.2. Novel Variants Associated with Oral Cancer in the Taiwanese

**Figure 1:**
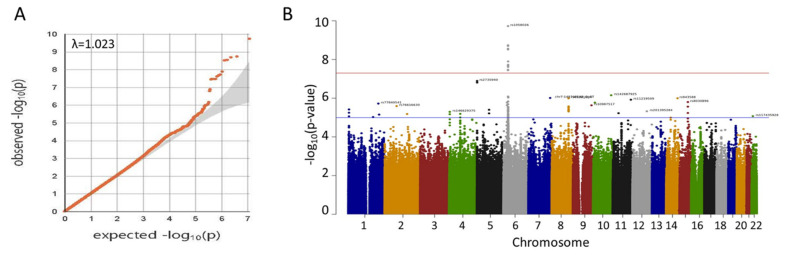
(**A**) Quantile–quantile plot of observed *p*-values for associations. The data indicate only a small amount of population inflation (λ = 1.023). (**B**) Manhattan plot of genome-wide *p*-values for associations. Red line indicates *p* = 5 × 10^−8^. Only SNPs in 6p21 reach genome-wide significance.

**Figure 2:**
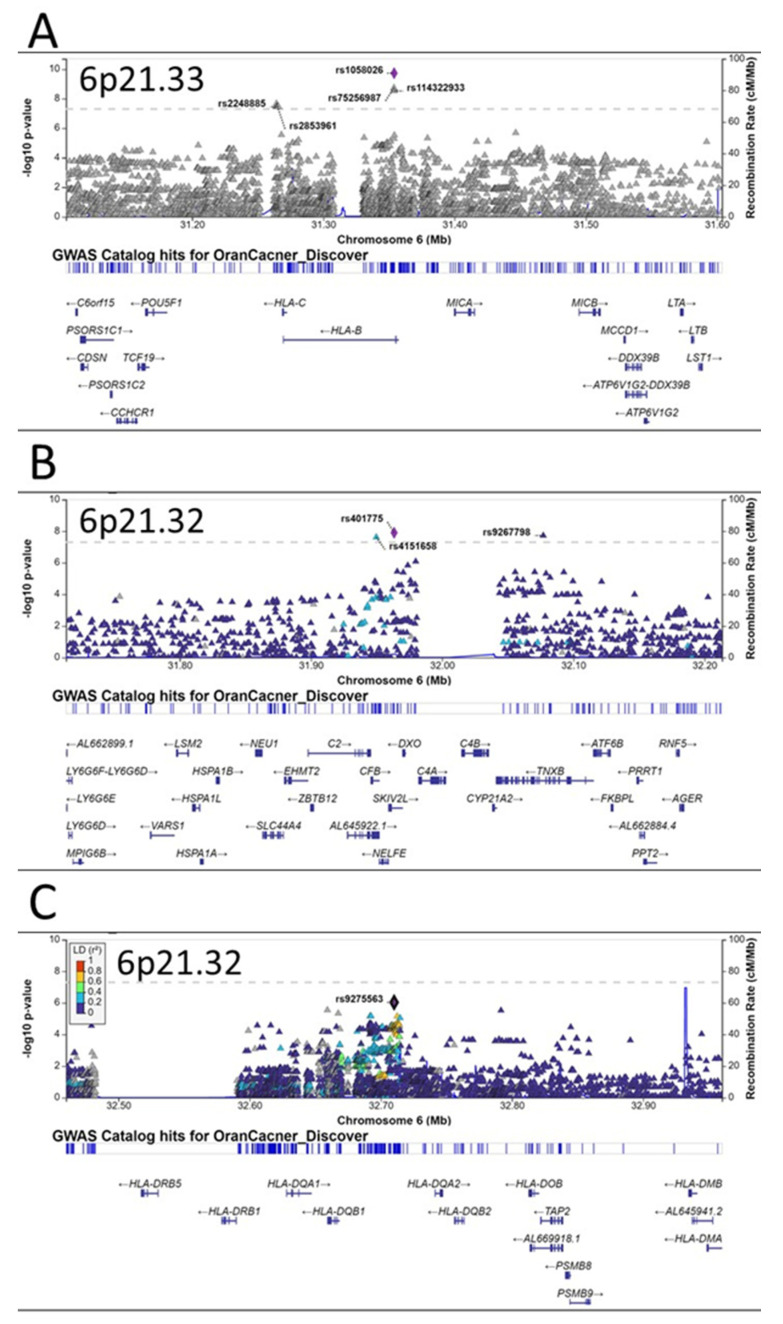
Regional plots of associations in 6p21. Three independent susceptibility loci for oral cancer are observed in 6p21 region (**A**–**C**). The *y*-axis presents the −log_10_ *p*-values of the SNPs and the *x*-axis presents the corresponding chromosomal position of each SNP. The genomic locations of genes within the regions were annotated from the UCSC Genome Browser.

A quantile–quantile plot of the observed versus expected χ^2^ test statistics did not show a large deviation from what was expected by chance (inflation factor λ = 1.023; Figure 1A). Fifteen variants on chromosome 6p21 were associated with oral cancer with genome-wide significance (*p* < 5 × 10^−8^) (Figure 1B, Table 2), twelve of which were highly linked and located in or near the HLA-B gene on 6p21.33 (Figure 2A). The other three, including one small insertion and two SNPs, were in the CFB, SKIV2L, and TNXB genes, respectively, on 6p21.32 (Figure 2B). The CFB and SIKV2L SNPs were in moderate linkage, and the TNXB SNP was not linked to the other two.

**Figure 3:**
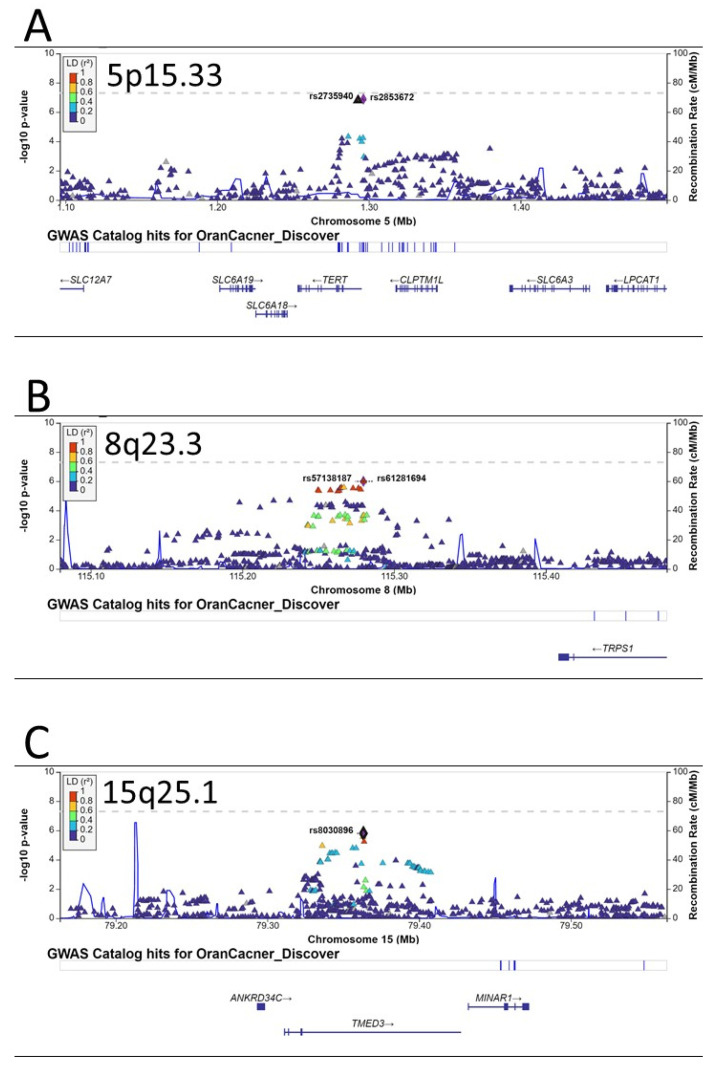
Regional plots of associations for regions of 5p15.33 (**A**), 8q23.3 (**B**), and 15q25.1 (**C**). The *y*-axis presents the −log_10_ *p*-values of the SNPs and the *x*-axis presents the corresponding chromosomal position of each SNP. The genomic locations of genes within the regions were annotated from the UCSC Genome Browser.

Besides these three independent loci on 6p21, there were several other promising loci that were close to having genome-wide significance, including another independent locus on 6p21.33 (HLA-DQ gene cluster, Figure 2C), the TERT-CLMPT1L locus on 5p15.33 (Figure 3A), a locus near the TRPS1 gene on 8q23.3 (Figure 3B), and the TMED3 gene on 15q25.1 (Figure 3C).

### 2.3. Common Variants Associated with Oral Cancer Validated among Various Populations

Previous GWAS in populations of European descent identified nine susceptibility loci for oral cancer. We queried these loci in our population. The consistent loci included rs1229984 in the ADH1B gene on 4q23, rs928674 in the LAMC3 gene on 9q34.12, and several loci on 6p21.32 and 6p21.33 (Table 3). For those loci that were close to genome-wide significance in previous GWAS, the TERT-CLMPT1L locus on 5p15.33 was highly consistent and the lead SNP was rs7726159 in the TERT gene (OR = 1.19, *p* = 2.18 × 10^−7^ in previous GWAS and OR = 1.16, *p* = 6.40 × 10^−5^ in our population) (Table 4).

## 3. Discussion

In this study, we performed the first GWAS of oral cancer in Taiwan. We confirmed two previously reported 6p21.33 and 6p21.32 HLA loci, and found two additional independent 6p21.33 loci for oral cancer in the Taiwanese. We also confirmed the previously reported oral cancer susceptibility loci at the TERT-CLMPT1L locus on 5p15.33, thet 4q23 ADH1B locus, and the LAMC3 locus on 9q34.12 in Taiwanese populations. We found suggestive novel susceptibility loci near the TPRS1 gene on 8q23.3 and in the TMED3 gene on 15q25.1.

The most notable finding of this study is the multiple independent oral cancer susceptibility loci spanning the 6p21.3 regions, which contain the human leukocyte antigen (HLA) gene clusters. The HLA system plays essential roles in innate and adaptive immune responses [29]. The HLA system consists of three regions: the class I region encodes HLA-A, -B, and -C; the class II region encodes HLA-DR, -DQ, and –DP; and the class III region genes code for proteins of the complement system and the TNF family members. The functions of class I and class II molecules are to bind intracellular and extracellular peptide antigens and present them to antigen-specific T lymphocytes. Peptide antigens associated with HLA class I molecules are recognized by CD8+ T cells and those associated with HLA class II molecules are recognized by CD4+ T cells [30]. The 6p21.3 is the most gene-dense region in the human genome and the HLA genes are the most polymorphic in the human genome [31]. Previous GWAS have reported genetic variants in various HLA genes as susceptibility loci for many human diseases, including a few cancers such as lung cancer [32], liver cancer [33], cervical cancer [34], colorectal cancer [35], leukemia [36], lymphoma [37,38], and different subtypes of head and neck cancers [23,27,28,39]. Specifically, for oral cancer, previous GWAS identified three loci in the HLA region, including rs3828805 (chromosome 6 position 32636120) [27] and rs3135001 (6:32670136) in HLA-DQB1 (6p21.32) [28] and rs1265081 (6:31111675) in CCHCR1 (6p21.33) [28]. Recently, Ji et al. found that two independent SNPs in the 6p21.33 regions were associated with altered oral cancer risks in a Chinese population: rs2524182 (6:31130593) in TRIM39-RPP21-HLA-E and rs3131018 (6:31143582) in PSORS1C3-TCF19 [40]. In our current study, we found many SNPs in 6p21 that reached genome-wide or borderline genome-wide significance in their associations with oral cancer in Taiwan, covering multiple HLA genes (Table 2, Figure 2), further supporting the important roles of the HLA system in oral cancer etiology.

For other GWAS-identified oral cancer susceptibility loci in European populations, we were able to replicate the loci on 5p15.33, the 4q23 ADH1B locus, and the LAMC3 locus on 9q34.12 (Table 3). The 5p15.33 region encompassing the TERT-CLPTM1L genes has been associated with the risks of at least 11 different cancers, including lung, prostate, breast, pancreatic, bladder, esophageal, endometrial, gastric, and head and neck cancers, glioma, and melanoma [28,32,41,42,43,44,45,46]. Multiple mechanisms, including telomere structure, epigenetic modification, transcriptional regulation, and apoptosis, have been suggested to explain the associations of the TERT-CLPTM1L locus and cancer susceptibility [47,48]. Chromosome 4q23 contains a cluster of alcohol dehydrogenase (ADH) genes and several SNPs in different ADH genes have been identified as susceptibility loci for oral cancer in European populations (Table 3) and other populations [49]. The best-studied SNP is rs1229984, a missense SNP at codon 47 (Arg47His) of the ADH1B. The A allele (coding for His) is about 40 times more active in metabolizing alcohol and is associated with a reduced risk of oral cancer. The frequency of the A (His) allele is only ~5% in European populations but reaches ~80% in East Asians. We used the predominant A (His) allele as the reference group, and those with the less active G (Arg) allele had 20% (OR = 1.20, 95% CI, 1.06–1.35, *p* = 0.003), consistent with literature reports in Europeans and Asians [49].

We also found potential novel susceptibility loci at 8q23.3 near the TRPS1 gene. TRPS1 is an atypical member of the GATA transcriptional factor family, exhibiting transcriptional repression by interacting with corepressors [50,51,52]. Recent studies have suggested that TRPS1 is overexpressed in several cancers and can act as an oncogenic driver through various mechanisms [53], such as driving heterochromatic origin refiring and genome amplifications [54], controlling the cell-cycle progression [55], promoting epithelial-to-mesenchymal transition [56], promoting angiogenesis [57], and causing epigenetic alterations (DNA methylation and histone acetylation) [50,58,59]. There has been no report of TRPS1 in oral cancer. Future studies are warranted to investigate the role of TRPS1 in oral carcinogenesis. Another novel oral cancer susceptibility locus is in the TMED3 gene on 15q25.1. TMED3 is a transmembrane protein and plays an important function in vesicular transport and innate immunity [60]. Several recent studies have shown an increased expression of TMED3 in a number of cancers and TMED3 promotes the carcinogenesis of liver, breast, colorectal, lung, and endometrial cancer as well as glioma and osteosarcoma [61,62,63,64,65,66,67]. Biologically, TMED3 can activate IL-11/STAT3 and Wnt/beta-catenin signaling pathways [61,62]. The role of TMED3 in oral carcinogenesis remains to be investigated.

Whether these oral susceptibility loci in the Taiwanese are consistent in other Asian populations are largely unknown. A recent study in China [40] used Human Exome BeadChip (~240K mostly nonsynonymous coding variants), but the final analyzable SNPs were only ~63K because most of the SNPs were monomorphic. They found two independent oral cancer susceptibility loci in the 6p21.33 regions: rs2524182 in TRIM39-RPP21-HLA-E and rs3131018 168 in PSORS1C3-TCF19 [40], consistent with our data. Other regions remain to be investigated. The data in India were more limited. Only one early pilot GWAS using human CNV370k BeadChip in only 55 cases and 92 controls was published [68]. Due to its small sample size, none of the reported European and Taiwanese oral cancer susceptibility loci were among the top hits in that study. The genetic susceptibility loci to oral cancer in India warrant further investigation.

The findings from our study not only shed significant insight into the biology of oral cancer etiology in Taiwan, but also have an important clinical and public health impact. The identified susceptibility genes may become potential preventive and/or therapeutic targets. For example, strategies that improve immune response, telomere maintenance, and alcohol metabolism may prevent oral cancer development given the importance of relevant genes in these pathways in oral cancer susceptibility. Another potential translational application is to use multiple genetic susceptibility loci to develop a polygenic risk score (PRS) for each person. This PRS can be integrated with environmental exposures such as smoking, drinking, and betel nut chewing to identify individuals at the highest risk of developing oral cancer, who then would be subjected to targeted cancer prevention and screening.

This is the first GWAS of oral cancer in Taiwan. We had the largest oral cancer cases and controls in any association studies of oral cancer to date. We found both common and unique oral cancer susceptibility loci in the Taiwanese as compared to European populations. Our large sample size enabled the unequivocal confirmation of HLA regions as the most prominent oral cancer susceptibility loci in Taiwan. We also confirmed the susceptibility loci at 5p15.33, the 4q23 ADH1B locus, and the LAMC3 locus on 9q34.12. We found novel suggestive susceptibility loci near the *TPRS1* gene on 8q23.3 and in the *TMED3* gene on 15q25.1.

## 4. Materials and Methods

### 4.1. Study Population and Data Collection

The study participants were part of the China Medical University Hospital (CMUH) Precision Medicine Project, a systemic effort initiated in 2018 to recruit subjects and collect biospecimens from all patients who come to CMUH for medical visits [69,70]. More than 170,000 subjects have been enrolled to date. The recruitment and sample collection procedures were approved by the ethical committees of CMUH (CMUH107-REC3-058 and CMUH110-REC3-005). Each participant signed an informed consent form and provided blood samples. Clinical information was abstracted from the electronic medical records (EMRs) of the CMUH. A total of 1529 oral cancer patients (ICD-10-CM Diagnosis Code C00 to C06) and 44,572 controls (without a history of any cancer) were included in the study.

### 4.2. Genotyping and Imputation

The whole genome SNP genotyping using the Affymetrix genome-wide human SNP array 6.0 chip was performed according to the manufacturer’s protocol [68]. We excluded samples and SNPs with genotyping call rates of <90%. We filtered out SNPs with a Hardy–Weinberg equilibrium *p*-value of <10^−6^, and a minor allele frequency (MAF) < 10^−4^. We excluded SNPs on sex chromosomes. Genotype imputation was performed as we recently described [69]. Briefly, we first constructed a population-specific reference panel by using whole genome sequencing data (1463 individuals) from the Taiwan Biobank (TWB). We used four algorithms (IMPUTE2, IMPUTE4, IMPUTE5, and Beagle5.2) and two reference panels (TWB and East Asian participants of the 1000 Genomes Project) to perform genotype imputation and found Beagle5.2 exhibited the fastest calculation speed, smallest storage space, highest specificity, and highest number of high-quality variants (15,277,414). The Beagle5.2 imputations were performed using its default parameters, except for the effective population size (20,000) and the buffer region (500,000 bases). The accuracy of the imputation result was measured using BCFtools gtcheck [71] to assess the concordance rate between the imputed genotypes and the WGS data. The imputation accuracy was 98.75% by Beagle5.2.

### 4.3. Statistical Analysis

For the participants’ characteristics, continuous data were presented as the means with standard deviation, and categorical data were presented as proportions. We used t-tests to compare the mean values of continuous variables and chi-squared tests to compare the frequencies of categorical variables between the cases and controls. The association of each SNP with the risk of oral cancer was analyzed using an additive model in the logistic regression analysis with PLINK V.1.90 [72]. To control for population structure, we performed principal component analysis (PCA) in EIGENSTRAT and adjusted significant principal components (PC) associated with the cancer status in unconditional logistic regression analysis, together with demographic variables including age and gender when estimating odds ratio (OR) and 95% confidence interval (CI). A genome-wide significance level was set at 5 × 10^−8^.

## Figures and Tables

**Table 1:** Selected characteristics of the study population.

Variables	Cases, N = 1529	Controls, N = 44,572
Age, mean (SD)	55 (11.5)	54 (14.5)
Sex (N, %)		
Male	1328 (86.9)	34,616 (77.7)
Female	201 (13.1)	9956 (22.3)

**Table 2:** Significant variants (*p* < 5 × 10^−8^) associated with oral cancer in the Taiwanese.

CHROM	POS	ID	Gene	REF	ALT	OR (95% CI)	*p*
6	31263915	rs2524113	TCF19/HLA-C	T	G	1.32 (1.2–1.46)	3.40 × 10^−8^
6	31264003	rs2524112	TCF19/HLA-C	A	G	1.32 (1.2–1.46)	3.40 × 10^−8^
6	31264212	rs2853961	TCF19/HLA-C	G	A	1.32 (1.2–1.46)	2.12 × 10^−8^
6	31264470	rs2844624	TCF19/HLA-C	T	C	1.32 (1.2–1.46)	3.40 × 10^−8^
6	31264813	rs2524107	TCF19/HLA-C	C	T	1.32 (1.2–1.46)	3.40 × 10^−8^
6	31265454	rs2246085	TCF19/HLA-C	A	T	1.32 (1.2–1.46)	3.40 × 10^−8^
6	31265793	rs2248885	HLA-C/CLIC1	C	T	1.32 (1.2–1.46)	3.54 × 10^−8^
6	31353774	rs75456009	HLA-C/CLIC1	A	G	1.32 (1.2–1.44)	2.77 × 10^−9^
6	31353782	rs74615740	HLA-C/CLIC1	C	A	1.32 (1.21–1.45)	1.98 × 10^−9^
6	31353786	rs75256987	HLA-C/CLIC1	A	C	1.32 (1.21–1.45)	1.84 × 10^−9^
6	31353800	rs114322933	HLA-C/CLIC1	C	T	1.32 (1.2–1.45)	3.01 × 10^−9^
6	31353908	rs1058026	HLA-B	A	C	1.34 (1.23–1.47)	1.87 × 10^−10^
6	31949780	rs4151658	CFB	C	CAT	1.37 (1.23–1.53)	2.41 × 10^−8^
6	31963360	rs401775	SKIV2L	T	C	1.34 (1.21–1.49)	1.25 × 10^−8^
6	32077057	rs9267798	TNXB	G	C	0.76 (0.69–0.84)	1.88 × 10^−8^

**Table 3:** Replication of previous GWAS-identified oral cancer susceptibility loci in Taiwan population.

Region	SNP	Chrom:Position	Gene	Allele	EAF Cases/Controls	OR	*p*	OR (95% CI)	*p*
Lesseur et al. Nature Genetics, 2016								(Taiwanese)	(Taiwanese)
OC and OPC									
4q23	rs1229984	4:100239319	ADH1B	G/A	0.03/0.06	0.56	2.29 × 10^−15^	1.20 (1.06–1.35)	0.00335
6p21.32	rs3828805	6:32636120	HLA-DQB1	C/T	0.75/0.72	1.28	3.35 × 10^−13^	0.75 (0.58–0.96)	0.0249
10q26.13	rs201982221	10:126157446	LHPP	Ins/Del	0.03/0.02	1.67	1.58 × 10^−9^	NA	NA
11p15.4	rs1453414	11:5829084	OR52N2–TRIM5	A/C	0.23/0.20	1.19	4.78 × 10^−8^	NA	NA
OC									
2p23.3	rs6547741	2:27855924	GPN1	G/A	0.50/0.54	0.83	3.97 × 10^−8^	1.00 (0.84–1.20)	0.98
4q23	rs1229984	4:100239319	ADH1B	G/A	0.03/0.06	0.57	1.09 × 10^−9^	1.20 (1.06–1.35)	0.003
5p15.33	rs10462706	5:1343794	CLPTM1L	C/T	0.12/0.15	0.74	5.54 × 10^−10^	0.93 (0.83–1.04)	0.234
9p21.3	rs8181047	9:22064465	CDKN2B-AS1	G/A	0.29/0.24	1.24	3.80 × 10^−9^	1.17 (0.99–1.37)	0.064
9q34.12	rs928674	9:133952024	LAMC3	A/G	0.14/0.12	1.33	2.09 × 10^−8^	0.75 (0.63–0.90)	0.002
Shete et al. Cancer Research, 2020									
HNSCC									
6p22.1	rs259919	6:30025503	ZNRD1-AS1	A/G	0.34/0.31	1.15 (1.06–1.25)	2.96 × 10^−9^	0.94 (0.83–1.07)	0.353
6p21.33	rs1265081	6:31111675	CCHCR1	C/A	0.47/0.5	0.85 (0.79–0.92)	3.75 × 10^−10^	0.95 (0.85–1.07)	0.414
6p21.32	rs3135001	6:32670136	HLA-DQB1	T/C	0.21/0.25	0.76 (0.69–0.83)	1.44 × 10^−16^	0.76 (0.59–0.97)	0.03
OC									
5p15.33	rs10462706	5:1343794	CLPTM1L	T/C	0.12/0.15	0.72 (0.60–0.88)	7.87 × 10^−11^	0.93 (0.83–1.05)	0.234
6p21.32	rs1049055	6:32634387	HLA-DQB1	C/T	0.23/0.27	0.76 (0.66–0.89)	2.96 × 10^−9^	0.71 (0.55–0.92)	0.009
Ji et al. J Med Genet, 2022									
HNSCC									
6p22.1	rs2517611	6:30169327	TRIM26	A/G	0.15/0.11	1.32 (1.18–1.47)	7.45 × 10^−7^	1.22 (0.96–1.55)	0.096
6p21.33	rs2524182	6:31130593	TRIM39-RPP21, HLA-E	A/T	0.37/0.31	1.27 (1.17–1.37)	1.76 × 10^−9^	1.04 (0.90–1.21)	0.562
6p21.33	rs3131018	6:31143582	PSORS1C3-TCF19	C/A	0.22/0.30	0.77 (0.71–0.83)	2.17 × 10^−10^	0.84 (0.75–0.94)	0.004
9p21.3	rs1063192	9:22003367	CDKN2B	A/G	0.24/0.19	1.33 (1.2–1.45)	4.10 × 10^−12^	1.11 (0.96–1.28)	0.166
McKay et al. Plos Genetics, 2011									
UADT Cancer									
4q23	rs1229984	4:99318162	ADH1B	C/T	EAF: 0.05	0.64 (0.59–0.71)	1.00 × 10^−20^	1.20 (1.06–1.35)	0.00335
4q23	rs971074 (or rs1573496)	4:99420704	ADH7	G/C	EAF: 0.12	0.75 (0.70–0.80)	9.00 × 10^−19^	1.22 (1.02–1.44)	0.0264
4q23	rs1789924 (or rs698)	4:99353129	ADH1C	T/C	EAF: 0.39	1.12 (1.07–1.17)	3.00 × 10^−7^	1.13 (0.93–1.36)	0.215
12q24	rs4767364	12:112083644	ALDH2	G/A	EAF: 0.27	1.13 (1.08–1.18)	2.00 × 10^−8^	0.89 (0.71–1.10)	0.283
4q21	rs1494961	4:83453327	HEL308	T/C	EAF: 0.49	1.12 (1.08–1.17)	1.00 × 10^−8^	0.94 (0.83–1.06)	0.33

**Table 4:** Borderline significant SNPs (5 × 10^−8^ < *p* < 1 × 10^−7^) in Europeans and their associations with oral cancer in Taiwanese.

Region	SNP ID	Chr:Position	Gene	Effect Allele	Other Allele	Europeans		Taiwanese	
						OR	*p*	OR	*p*
2p23.3	rs4665986	2:27755168		A	G	0.84	1.75 × 10^−7^	1.114	0.030
2p23.3	rs4665381	2:27757150		C	A	0.84	1.30 × 10^−7^	1.101	0.054
2p23.3	rs1972669	2:27767107		G	T	0.84	1.50 × 10^−7^	1.101	0.054
2p23.3	rs4493210	2:27768072		T	G	0.84	1.08 × 10^−7^	1.101	0.053
2p23.3	rs1080060	2:27768808		A	T	0.84	1.08 × 10^−7^	1.101	0.054
2p23.3	rs2384626	2:27773648		G	A	0.84	2.93 × 10^−7^	1.101	0.054
2p23.3	rs1820297	2:27774924		G	A	0.84	6.23 × 10^−8^	1.096	0.065
2p23.3	rs6750943	2:27777904		G	T	0.84	9.17 × 10^−8^	1.096	0.065
2p23.3	rs6761095	2:27778010		T	G	0.84	1.78 × 10^−7^	1.096	0.065
2p23.3	rs6706610	2:27778091		A	G	0.84	6.41 × 10^−8^	1.096	0.065
2p23.3	rs6547709	2:27783418		A	G	0.84	8.93 × 10^−8^	1.094	0.068
2p23.3	rs12714204	2:27787540		C	T	0.84	1.66 × 10^−7^	1.094	0.068
2p23.3	rs4260197	2:27794150		G	C	0.84	1.70 × 10^−7^	1.095	0.068
2p23.3	rs10205364	2:27794233		T	C	0.84	1.20 × 10^−7^	1.095	0.068
2p23.3	rs10165098	2:27796526		T	C	0.84	8.30 × 10^−8^	1.095	0.068
2p23.3	rs1919125	2:27801403	C2orf16	G	C	0.84	1.23 × 10^−7^	1.095	0.066
2p23.3	rs1919126	2:27801418	C2orf16	C	A	1.19	1.28 × 10^−7^	1.095	0.066
2p23.3	rs13026621	2:27807624	ZNF512	A	G	0.84	8.99 × 10^−8^	1.091	0.079
2p23.3	rs4665995	2:27813827	ZNF512	G	T	0.84	9.66 × 10^−8^	1.091	0.079
2p23.3	rs4665996	2:27814343	ZNF512	C	T	0.84	9.96 × 10^−8^	1.091	0.079
2p23.3	rs6738089	2:27815278	ZNF512	G	A	0.84	2.14 × 10^−7^	1.091	0.079
2p23.3	rs4665997	2:27816989	ZNF512	C	T	0.84	1.53 × 10^−7^	1.094	0.072
2p23.3	rs7604798	2:27824940	ZNF512	C	T	0.84	1.81 × 10^−7^	1.089	0.087
2p23.3	rs62138969	2:27828292	ZNF512	A	G	0.84	2.10 × 10^−7^	1.088	0.090
2p23.3	rs6547734	2:27830990	ZNF512	T	G	0.84	1.52 × 10^−7^	1.088	0.090
2p23.3	rs6718128	2:27833752	ZNF512	T	C	0.84	3.19 × 10^−7^	1.088	0.091
2p23.3	rs4665999	2:27834444	ZNF512	C	T	0.84	1.04 × 10^−7^	1.088	0.090
2p23.3	rs11127071	2:27838058	ZNF512	A	C	0.84	1.06 × 10^−7^	1.077	0.140
2p23.3	rs6737921	2:27839107	ZNF512	C	T	0.84	1.08 × 10^−7^	1.076	0.141
2p23.3	rs6756238	2:27841305	ZNF512	A	G	0.84	1.11 × 10^−7^	1.074	0.153
2p23.3	rs1528404	2:27846603		C	T	0.84	1.56 × 10^−7^	1.062	0.312
2p23.3	rs1528403	2:27846645		G	C	0.85	3.86 × 10^−7^	1.059	0.336
2p23.3	rs2384654	2:27847364		T	C	0.84	1.78 × 10^−7^	1.059	0.335
2p23.3	rs4666005	2:27855239	GPN1	G	A	0.84	2.14 × 10^−7^	1.072	0.237
2p23.3	rs2384659	2:27856793	GPN1	A	G	0.84	6.71 × 10^−8^	1.072	0.236
2p23.3	rs4666009	2:27857992	GPN1	A	T	0.84	1.75 × 10^−7^	1.073	0.235
2p23.3	rs56001553	2:27859926	GPN1	T	C	1.19	2.53 × 10^−7^	1.068	0.265
5p15.33	rs7726159	5:1282319	TERT	A	C	1.19	2.18 × 10^−7^	1.161	6.40 × 10^−5^
5p15.33	rs60622800	5:1309904	MIR4457	A	G	0.84	6.56 × 10^−8^	1.141	0.004
5p15.33	rs6554758	5:1310152	MIR4457	A	G	0.84	6.24 × 10^−8^	1.141	0.004
5p15.33	rs380286	5:1320247	CLPTM1L	A	G	1.18	3.16 × 10^−7^	1.172	0.001
5p15.33	rs13178866	5:1323212	CLPTM1L	A	G	1.19	8.00 × 10^−8^	1.149	0.002
5p15.33	rs466502	5:1325767	CLPTM1L	G	A	1.19	1.31 × 10^−7^	1.152	0.002
5p15.33	rs465498	5:1325803	CLPTM1L	G	A	1.19	5.48 × 10^−8^	1.156	0.001
5p15.33	rs452384	5:1330840	CLPTM1L	G	A	1.19	5.74 × 10^−8^	1.156	0.001
5p15.33	rs370348	5:1331219	CLPTM1L	G	A	1.19	5.83 × 10^−8^	1.149	0.002
9p21.3	rs944800	9:22050898	CDKN2B-AS1	A	G	1.21	7.42 × 10^−8^	1.059	0.342
9p21.3	rs2383205	9:22060935	CDKN2B-AS1	A	G	1.20	2.55 × 10^−7^	1.036	0.542
9p21.3	rs1537378	9:22061614	CDKN2B-AS1	A	G	1.20	1.68 × 10^−7^	1.033	0.575
9p21.3	rs8181050	9:22064391	CDKN2B-AS1	G	A	1.20	1.74 × 10^−7^	1.028	0.634
9q34.12	rs3765566	9:133942766	LAMC3	G	A	1.31	2.28 × 10^−7^	0.931	0.307
9q34.12	rs7875478	9:133947180	LAMC3	T	A	1.32	1.23 × 10^−7^	0.932	0.312
9q34.12	rs7858204	9:133947345	LAMC3	A	G	1.32	1.20 × 10^−7^	0.930	0.295
9q34.12	rs10901348	9:133956465	LAMC3	A	G	1.31	7.12 × 10^−8^	1.062	0.270
9q34.12	rs72768533	9:133959518	LAMC3	G	C	1.31	6.82 × 10^−8^	0.981	0.783
9q34.12	rs72768534	9:133959740	LAMC3	C	G	1.31	6.77 × 10^−8^	0.981	0.785
9q34.12	rs77452476	9:133962573	LAMC3	G	T	1.29	5.97 × 10^−8^	0.986	0.748
9q34.12	rs11791030	9:133971049	LOC105376298	G	T	1.31	1.68 × 10^−7^	0.985	0.834
15q21.2	rs12910284	15:49785916	LOC105370811	G	A	1.19	4.28 × 10^−7^	1.029	0.438
15q21.2	rs10851478	15:49829019	LOC105370811	G	A	1.19	3.47 × 10^−7^	1.030	0.419
6p21.33	rs3132451	6:31582025	AIF1	C	G	1.25	4.59 × 10^−7^	0.866	0.029
6p21.32	rs9271378	6:32587300		G	A	1.19	2.05 × 10^−7^	1.017	0.694
6p21.32	rs17612852	6:32620572	HLA-DQA1	A	G	1.23	4.21 × 10^−7^	0.929	0.078
6p21.32	rs4713570	6:32626040		T	C	1.25	1.03 × 10^−7^	0.831	0.004
6p21.32	rs1049213	6:32627773	HLA-DQB1	G	A	1.24	4.65 × 10^−7^	0.910	0.018
6p21.32	rs3828805	6:32636120	HLA-DQB1	C	T	1.23	2.83 × 10^−7^	0.910	0.217
6p21.32	rs3135002	6:32668439		C	A	1.27	1.71 × 10^−7^	0.887	0.132
